# Prevalence of overweight in children and adolescents with attention deficit hyperactivity disorder and autism spectrum disorders: a chart review

**DOI:** 10.1186/1471-2431-5-48

**Published:** 2005-12-21

**Authors:** Carol Curtin, Linda G Bandini, Ellen C Perrin, David J Tybor, Aviva Must

**Affiliations:** 1 Eunice Kennedy Shriver Center, University of Massachusetts Medical School, Waltham, MA, USA; 2 Division of Developmental-Behavioral Pediatrics, Tufts-New England Medical Center, Boston, MA, USA; 3 the Department of Public Health and Family Medicine, Tufts University School of Medicine, Boston, MA, USA

## Abstract

**Background:**

The condition of obesity has become a significant public health problem in the United States. In children and adolescents, the prevalence of overweight has tripled in the last 20 years, with approximately 16.0% of children ages 6–19, and 10.3% of 2–5 year olds being considered overweight. Considerable research is underway to understand obesity in the general pediatric population, however little research is available on the prevalence of obesity in children with developmental disorders. The purpose of our study was to determine the prevalence of overweight among a clinical population of children diagnosed with attention deficit hyperactivity disorder (ADHD) and autism spectrum disorders (ASD).

**Methods:**

Retrospective chart review of 140 charts of children ages 3–18 years seen between 1992 and 2003 at a tertiary care clinic that specializes in the evaluation and treatment of children with developmental, behavioral, and cognitive disorders. Diagnostic, medical, and demographic information was extracted from the charts. Primary diagnoses of either ADHD or ASD were recorded, as was information on race/ethnicity, age, gender, height, and weight. Information was also collected on medications that the child was taking. Body mass index (BMI) was calculated from measures of height and weight recorded in the child's chart. The Center for Disease Control's BMI growth reference was used to determine an age- and gender-specific BMI z-score for the children.

**Results:**

The prevalence of at-risk-for-overweight (BMI >85th%ile) and overweight (BMI > 95th%ile) was 29% and 17.3% respectively in children with ADHD. Although the prevalence appeared highest in the 2–5 year old group (42.9%ile), differences among age groups were not statistically significant. Prevalence did not differ between boys and girls or across age groups (all p > 0.05). For children with ASD, the overall prevalence of at-risk-for-overweight was 35.7% and prevalence of overweight was 19%.

**Conclusion:**

When compared to an age-matched reference population (NHANES 1999–2002), our estimates indicate that children with ADHD and with ASD have a prevalence of overweight that is similar to children in the general population.

## Background

The condition of obesity has become a significant public health problem in the United States. Since 1980, the prevalence of obesity in adults has almost doubled, with current estimates of obesity present in over 35% of adults [1]. In children and adolescents, the prevalence of overweight has tripled in the last 20 years, with approximately 16.0% of children ages 6 through 19, and 10.3% of 2–5 year olds being considered overweight [1]. It should be noted that the terminology used to describe the condition of obesity differs between adults and children. For children, an expert committee on pediatric obesity [2] has recommended that the following terms be used to classify BMI (body mass index) cut-off points: children who have a BMI greater than or equal to the 85^th ^percentile ( ≥ 85%) are referred to as *at-risk-for-overweight*; children whose BMI is at or above the 95^th ^percentile ( ≥ 95%) are referred to as *overweight*. The term *obesity *is avoided in the categorization of weight status for children, which reflects the fact that BMI is not a direct measure of fatness and that mislabeling of a child as obese is potentially harmful [2].

The reasons for the alarming increase in prevalence of overweight in children are not well understood, but changes in demographics, family structure, lifestyle choices, decreases in physical activity, greater accessibility to food and increased portion sizes, as well as cultural/media influences are assumed to play a role [3]. The consequences of childhood obesity are significant; childhood obesity is associated with an increased risk for type 2 diabetes, orthopedic problems, sleep apnea, elevated cardiovascular risk factor levels, and menstrual irregularities [4]. Many overweight children already exhibit impaired glucose tolerance [5]. The likelihood of childhood obesity persisting into adulthood is increased with greater severity and younger age of onset [6].

Some research has been conducted to examine the prevalence of obesity in children with developmental disabilities, particularly in individuals with Down syndrome and Prader-Willi syndrome who are reported to have a higher prevalence of overweight than in the general population [7-9]. However, few studies have been undertaken to assess the prevalence of overweight in children with other developmental disorders, such as attention deficit hyperactivity disorder (ADHD) and autism spectrum disorders (ASD). Our clinical observations suggest that these children may be at increased risk for overweight due to fewer opportunities to engage in structured physical activity, social isolation, increased sedentary behavior such as television viewing or computer time, and unusual dietary patterns.

Attention Deficit Hyperactivity Disorder (ADHD) is one of the most common neurodevelopmental/behavioral disorders of childhood. The core features of this disorder include developmentally inappropriate levels of inattention, impulsivity, distractibility, and motoric overactivity (or hyperactivity). These behaviors cause functional impairment that is commonly seen across settings, such as school, home, and with peers [10]. Children with ADHD have academic, behavioral, and social/emotional difficulties that frequently require psychopharmacologic, educational, and psychosocial interventions.

Autism is a neurologically-based developmental disorder which appears in childhood, usually by the age of 3 years. As defined by the Diagnostic and Statistical Manual of Mental Disorders, 4^th ^edition (DSM-IV), autism falls under the broad diagnostic category of Pervasive Developmental Disorders and is marked by impairments in communication, forming relationships/social interaction, and often behavioral control [10]. The diagnostic categories of autistic disorder, PDD-NOS and Asperger's syndrome are considered to constitute a "spectrum" of autism and share many characteristics such as delayed speech/language development, delayed or unusual play routines, and stereotypic behaviors (e.g., spinning, hand flapping), narrow ranges of interest, and a tendency to display rigid and/or perseverative behavioral repertoires. Thus, the term Autism Spectrum Disorders (ASD) is used to characterize the continuum of children with these difficulties.

The aim of this study was to determine the prevalence of overweight among children diagnosed with ADHD and Autism Spectrum Disorders (ASD) seen in a tertiary care clinic.

## Methods

We reviewed patient records from a tertiary care clinic at the Tufts-New England Medical Center in Boston, MA that specializes in the evaluation and treatment of children with developmental, behavioral, and cognitive disorders. We selected every 5^th ^chart from the files available at the clinic to enable us to obtain a representative sample of clinic patients. To select charts of children seen recently, we selected the charts of patients seen for initial visit or for follow-up between 2002 and 2003. Each chart was reviewed for the presence of the diagnosis of ADHD or ASD. If the chart did not contain this diagnosis, the next 5^th ^chart was chosen. Of the charts drawn that contained these diagnoses, we included only charts with complete information on height, weight, and age; this yielded a final total of 140 charts. Ninety-eight (98) charts belonged to children who had a diagnosis of Attention Deficit Hyperactivity Disorder (ADHD) and 42 charts belonged to children who had Autism Spectrum Disorder (ASD).

Diagnostic, medical, and demographic information was extracted from each chart. Patients seen at the clinic undergo comprehensive evaluations in which the child and his/her parent(s) are interviewed extensively for a detailed history that includes information about birth, medical, developmental, school, family, and psychosocial histories. The child's parents are asked to provide background records and school reports as well as to complete behavior rating scales. The child's teachers are also asked to complete behavior rating scales in order to confirm that symptoms are occurring in the school setting as well as at home. Each child undergoes a thorough physical, neurological, and developmental examination. In many cases, children are also seen for neuropsychological, speech/language and educational evaluations. The diagnoses of ADHD and ASD are made using DSM-IV criteria and additional testing data. For this chart review, primary diagnoses of either ADHD or ASD were recorded, as was information on race/ethnicity, date of birth, gender, height, and weight. We also collected information on medications that the child was taking. Children were seen at various intervals for follow-up visits, therefore we used data obtained from the child's initial visit at the clinic.

Body mass index (BMI) was calculated from measures of height and weight indicated in the child's chart, taken by a physician or nurse at the time of the first visit. The Center for Disease Control's BMI growth reference was used to determine an age- (to the nearest month) and gender-specific BMI z-score for the children. Age was calculated based on date of birth and the date of the visit. We used z-scores corresponding to the 85^th ^(*at-risk-for-overweight*) and 95^th ^percentiles (*overweight*) of BMI as cut points to classify overweight [11]. The study protocol was approved by the hospital's Institutional Review Board and exempted from informed consent.

Prevalence estimates by gender and age group were compared within diagnosis by chi-square or by Fisher's Exact test, for any analysis with a count in a cell of fewer than 5. Prevalence estimates and their 95% confidence intervals (CI) were used to compare our estimates to population estimates of prevalence based on the most recently available national data, the National Health and Nutrition Examination Survey 1999–2002 [1]. Where the 95% CI included the national estimates, we concluded that estimates did not differ. Statistical significance was deemed present when p < 0.05. Because the information obtained was at the child's initial visit, the data we gathered spread over a period of 11 years (1992–2003). Because there have been secular increases in the prevalence of obesity, using the NHANES 1999–2002 prevalence estimates for comparison constitutes a conservative approach.

## Results

### Children with ADHD

Child characteristics are presented in Table 1. Eighty-one percent (81%) of the children with ADHD were white, 7% were African American, 3% were Hispanic, 8% were Asian, and 1% were of other race/ethnicity. Eighty-one percent (81%) were boys and 19% were girls. The majority of children with ADHD (59.2%) were between 6–11 years of age.

**Table 1:** Demographic characteristics of age, gender and race/ethnicity of charts reviewed for children with ADHD and ASD at an ambulatory care clinic.

	**ADHD**	**ASD**
	N = 98	N = 42
**Age (y)**		
2–5	14.3% (14)	50% (21)
6–11	59.2% (58)	38% (16)
12–18	26.5% (26)	12% (5)
**Gender**		
Male	81% (79)	81% (34)
Female	19% (19)	19% (8)
**Race/Ethnicity**		
White	81% (79)	83.3% (35)
African American	7% (7)	0 (0)
Hispanic	3% (3)	4.8% (2)
Asian	8% (8)	9.5% (4)
Other race/ethnicity	1% (1)	2.4% (1)

( ) = no. of subjects

The prevalence of at-risk-for-overweight was 29% in children with ADHD (Table 2) and did not differ significantly between boys and girls (31% vs. 25.9%). The prevalence appeared highest in the 2–5 year old group (42.9%), but differences among age groups were not statistically significant.

**Table 2:** Prevalence (95% confidence interval) of at-risk-for-overweight ( ≥ 85 percentile)^1 ^and overweight ( ≥ 95 percentile) by age in children with ADHD and national estimates.

	NHANES 1999–2002*	**ADHD N = 98**
**AT RISK**		
2–5 yr	22.6%	42.9% (16.9, 68.8)
6–11 yr	31.2%	25.9% (14.6, 37.1)
12–19 yr	30.9%	30.8% (13.0, 48.5)
Overall	31.0%	29% (20.0, 38.0)
		
**OVERWEIGHT**		
2–5 yr	10.3%	21% (0.0, 42.3)
6–11 yr	15.8%	16% (6.6, 25.4)
12–19 yr	16.1%	19% (3.9, 34.0)
Overall	16.0%	17.3% (9.8, 24.8)

^1^CDC recommended cut-off points for at-risk-for overweight and overweight (11). *Hedley et al. JAMA 2004

The prevalence of overweight in children with ADHD was 17.3%. Prevalence estimates of boys and girls and across age groupings were not significantly different. When our estimates are compared to an age-matched reference population (NHANES 1999–2002) [12], children with ADHD have a prevalence of overweight that does not differ from children in the general population.

#### Medication use

Because the use of stimulant medication is common among children with ADHD and may influence weight stability, we examined the relationship of stimulant medication use to weight status (Table 3). Thirty-three percent (33%) of the 98 children with ADHD whose charts we reviewed were receiving stimulant medication at the initial visit. Of these children, 5 (16%) were considered at-risk-for-overweight and 2 (6.3%) were overweight. In comparison, 24 (36%) of the children who were not receiving stimulant medication were at-risk-for-overweight and 15 (23%) were classified as overweight. These differences are statistically significant (p < 0.05, Fisher's Exact test). Interestingly, none of the children taking stimulant medications were underweight (i.e., below the 15^th ^percentile), although 5 (16%) of the children *not *receiving stimulant medication were underweight.

**Table 3:** Prevalence of at-risk-for-overweight( ≥ 85^th ^%ile)^1^and overweight ( ≥ 95^th ^%ile) in children with ADHD receiving stimulants medication to those not receiving stimulant medication.

**Receiving stimulant medication**	**All Subjects**	**Underweight <15^th ^percentile**	**Healthy Weight 15^th ^to 85^th ^percentile**	**At Risk 85^th ^to 95^th ^percentile**	**Overweight >95^th ^percentile**
**YES**	33% (32)	0	78% (25)	16% (5)	6% (2)
**NO**	67% (66)	8% (5)	33% (22)	36% (24) *	23% (15) *
**Total**	100% (98)	5% (5)	48% (47)	30% (29)	17% (17)

**^1^CDC recommended cut-off points for at-risk-for overweight and overweight (11). **Fisher Exact Test (p < .05)

Small numbers of children with ADHD received other types of medications (Serotonin reuptake inhibitors (SSRI's) n = 2; anticonvulsants n = 1; anti-hypertensives n = 7; other antidepressants n = 1; and other medications n = 5). Because of the small sample sizes we were not able to examine these medications in relation to weight status.

### Children with Autism Spectrum Disorders (ASD)

Eighty-three percent (83%) of the children with an ASD diagnosis were white, 5% were Hispanic, 10% were Asian, and 2% were of other race/ethnicity (Table 1). Eighty-one percent (81%) were male. In this group, the overall prevalence of at-risk-for-overweight was 35.7% and prevalence of overweight was 19% (Table 4). When stratified by age, the prevalence of at-risk-for-overweight and overweight appears to be highest in the 12.0–17.9 year old group, however differences by age category were not significant.

**Table 4:** Prevalence (95% confidence interval) of at-risk-for-overweight ( ≥ 85 percentile)^1 ^and overweight ( ≥ 95 percentile) by age in children with Autism Spectrum Disorders

	*NHANES 1999–2000	**AUTISM SPECTRUM DISORDERS (ASD) N = 42**
**AT-RISK-FOR-OVERWEIGHT**		
2–5 yr	22.6%	23.8% (5.6, 42.0)
6–11 yr	31.2%	37.8% (14.0, 61.6)
12–19 yr	30.9%	80% (44.9, 100.0+)
Overall	31.0%	35.7% (21.2, 50.2)
		
**OVERWEIGHT**		
2–5 yr	10.3%	14.2% (0.0, 29.1)
6–11 yr	15.8%	18.8% (0.0, 37.9)
12–19 yr	16.1%	50% (6.1, 93.8)
Overall	16.0%	19.0% (7.1, 30.1)

^1^CDC recommended cut-off points for at-risk-for overweight and overweight (11). * Hedley et al. JAMA 2004

We were unable to conduct an analysis of medication use in relation to overweight because only a small number of children in the ASD sample were on medication, and the types of medication were quite variable.

## Discussion

Our data from this chart review suggest that as for the general population of children in the US, overweight represents a problem among children with ADHD and ASD. The prevalence of at-risk-for-overweight (BMI ≥ 85^th ^percentile) and overweight (BMI ≥ 95^th ^percentile) was 29% and 17.3% respectively in children with ADHD. For children with ASD, the overall prevalence of at-risk-for-overweight was 35.7% and prevalence of overweight was 19%.

The literature on obesity in children with ADHD is sparse. Historically, inquiries into weight status of children with ADHD have focused on the potential for growth suppression associated with the use of stimulant medication. More recently, however, some research has been conducted to examine the prevalence of overweight in children with ADHD. Anderson et al. [13] reported an association of increased relative weight among girls with ADHD but not boys in a community-based sample. Holtkamp et al. [14] evaluated a sample of 97 boys with ADHD in Germany to test the hypothesis that hyperactive boys would have a lower prevalence of obesity than an age-matched healthy male reference population. Contrary to expectations, they found that a significant number of subjects with ADHD had a BMI ≥ 90^th ^percentile (19.6%) and 7.2% had a BMI ≥ 97^th ^percentile using the higher International Obesity Task Force cut-offs points. Altfas et al. [15] conducted a chart review of 215 adults seen in a weight control clinic. Among these patients, 90% of whom were female, 27.4% had ADHD, 33.5% had symptoms and behaviors of ADHD but did not meet formal diagnostic criteria, and 39.1% did not have ADHD. All of the patients with ADHD were classified as having the inattentive type of the disorder. Of those patients with a BMI ≥ 40, 42.6% had ADHD. The authors noted that patients with ADHD were less successful at losing weight than those without ADHD. In a recent study of 26 children, hospitalized for obesity (BMI>85%) (mean age 13), Agranat-Meged et al. [16] found that 15 of these children had ADHD. Nine of these children had not been diagnosed prior to the study. Ten of the 13 boys and 5 of the 13 girls were found to have ADHD. Of the 15 children with ADHD, 9 had the combined type of ADHD and 6 had the inattentive type. Although this study suggests a relation between ADHD and obesity, the number of patients was relatively small and the study lacked a comparison group.

Although the criteria for overweight may differ among studies, our data and the reported data to date suggest that overweight is a problem among children with ADHD. Furthermore, the data reported by Altfas et al. and Agranat-Meged et al. underscore the importance of understanding and preventing the problem of overweight in children with ADHD, before they become adults.

The children in our study taking stimulant medication were half as likely to be overweight as those not on these medications however, none of the children in our sample who took stimulant medication were underweight. Reports in the literature of the effects of stimulant medication on weight status are equivocal; for example, one study found weight loss among children taking stimulant medication [17] whereas another found no weight deficits [18].

Only a few studies have reported data on weight status of children with ASD. In one recent report, Mouridsen et al. [19] examined the weight status of 117 young Danish children with autism. Body mass index (BMI) for males but not for females was significantly lower than an age-matched reference population. In Germany, a low BMI has been reported in 13 children with Asperger's syndrome [20]. In a large study of 20,031 Japanese children and adolescents with mental retardation (6–17 yrs) that included 413 children with autism, the prevalence of obesity was reported to be 22% in boys and 11% in girls. No data were available to assess the prevalence among different age groups and obesity was defined from measures of standard weight for height [21]. Another study of 140 Japanese children 7–18 years of age with autism revealed that 25% of the children were classified as obese [22]. The variability in the prevalence estimates among children with autism from different countries may be explained by different environmental factors that contribute to the development of overweight. In addition, different definitions of obesity, the diagnostic category, or mode of recruitment into the studies may contribute to observed differences. Nonetheless, these data support our findings that overweight is as significant a problem in children with autism as in the general population.

For children with ASD, unusual dietary patterns and decreased access to opportunities for physical activity may be factors that contribute to overweight. Rosser and Frey [23] report less time spent in moderate activity in children with ASD compared to children without ASD. Opportunities to engage in structured activities may be limited and may further decline with age for children with ASD. In our study, although the numbers in each age group are small, we observed a trend toward increasing prevalence of overweight with increasing age for children with ASD. Further research is needed to determine whether the prevalence of obesity in children with ASD increases with age and whether differences in eating and activity patterns contribute to overweight. Identification of specific environmental factors that are associated with increasing the risk for overweight (e.g., access to physical activity programs, time spent in sedentary behavior) are important areas of research that will help direct efforts at health promotion and disease prevention for this population of children.

Our study data were obtained from a chart review at a tertiary care clinic, hence, the data gathered represent children from a special population. Therefore, our findings may not be generalizable to the broader community of children with ADHD, and should be considered preliminary. Furthermore, because we relied on clinically derived diagnoses of ADHD and ASD rather than on objective, standardized measures, we are unable to determine the diagnostic homogeneity of the clinical charts reviewed. We chose to look at data at the initial visit, thus additional diagnostic information that may have surfaced on subsequent visits, such as the presence of depression, a known risk factor in ADHD was unavailable. Because depression is a frequent co-morbidity in children with ADHD and has been shown to be related to obesity [24-26] depression may have been a confounding factor. Thus, larger studies of children with ADHD and ASD adjusting for co-morbid conditions need to be carried out to elucidate further these findings. Finally, the number of charts reviewed was small, so our prevalence estimates lack a high degree of precision.

## Conclusion

As noted, data from this chart review suggest that just as overweight is a problem for the general population of children in the US, overweight also represents a problem among children with ADHD and ASD. The prevalence of at-risk-for-overweight (BMI ≥ 85^th ^percentile) and overweight (BMI ≥ 95^th ^percentile) was 29% and 17.3% respectively in children with ADHD. For children with ASD, the overall prevalence of at-risk-for-overweight was 35.7% and prevalence of overweight was 19%.

Despite noted limitations, our data suggest that the problem of overweight in children with ADHD and ASD is at least as significant as in the general population. Factors that contribute to the development of overweight or its maintenance in this population of children may be different than in children without these disorders, and therefore warrant further investigation. Furthermore, the burden of managing potential co-morbidities associated with obesity may be particularly problematic for children with developmental disorders and their families, and may limit options for living independently in adulthood. Therefore, this area of research warrants focused attention and effort.

## Competing interests

The author(s) declare that they have no competing interests.

## Authors' contributions

CC was a co-investigator and contributed to the design, data collection, and drafted the manuscript. LGB was a co-investigator and contributed to the design, data collection, and manuscript preparation. AM was the principal investigator of the study and contributed to the design, analytic plan, and manuscript preparation. DJT assisted with the statistical analysis. ECP is the medical director of the clinic where the study was conducted and collaborated on the manuscript preparation.

## Pre-publication history

The pre-publication history for this paper can be accessed here:


